# RON and c-Met facilitate metastasis through the ERK signaling pathway in prostate cancer cells

**DOI:** 10.3892/or.2022.8388

**Published:** 2022-08-18

**Authors:** Binbin Yin, Zhenping Liu, Yiyun Wang, Xuchu Wang, Weiwei Liu, Pan Yu, Xiuzhi Duan, Chunhua Liu, Yuhua Chen, Yurong Zhang, Xiaoyan Pan, Hangping Yao, Zhaoping Liao, Zhihua Tao

Oncol Rep 37: 3209–3218, 2017; DOI: 10.3892/or.2017.5585

Subsequently to the publication of the above article, the authors have discovered that the version of Fig. 5 included in the paper was an incorrect version, and that two pairs of data panels were inadvertently included in Fig. 6D (the data panels for the NC+migration and NC+HGF+U0126+invasion experiments for the PC3 cells, and the data panels for the NC+invasion and NC+HGF+U0126+invasion experiments for the DU145 cells) that contained overlapping data derived from the same source. These data were intended to represent the results obtained under different experimental conditions. Furthermore, the GAPDH control bands in Fig. 4A (DU145 cells) and the p-ERK1/2 bands in Fig. 6A (PC3 cells) were incorrectly chosen for these figures. After having consulted the original data, the authors discovered that unintended errors were made in assembling the data for these graphs. In uploading the corrected version of Fig. 5, Fig. 3C and D and Fig. 4C and D were adjusted accordingly.

The corrected versions of Figs. 3, 4, 5, and 6 are shown on the subsequent pages. The authors regret the errors that were made during the preparation of the published figures, and confirm that these errors did not affect the conclusions reported in the study. The authors are grateful to the Editor of *Oncology Reports* for allowing them the opportunity to publish a Corrigendum, and all the authors agree to this Corrigendum. Furthermore, they apologize to the readership for any inconvenience caused.

## Figures and Tables

**Figure 3.**
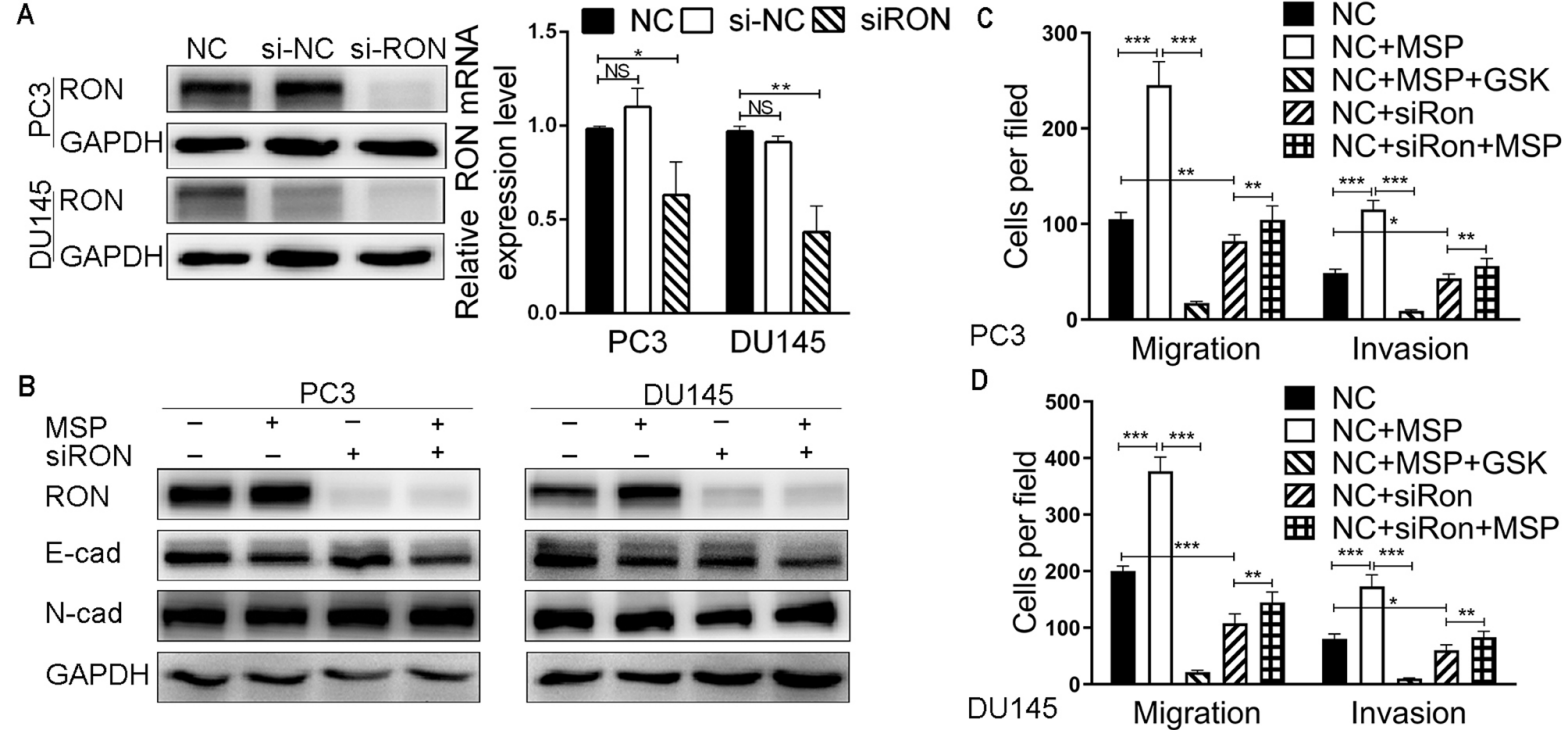
Functional role of RON in the migration and invasion of PCa cell lines. (A) Detection of siRNA-mediated knockdown of RON in PC3 and DU145 cells by western blotting and real-time PCR. (B) The expression of RON, E-cadherin and N-cadherin was determined by western blotting after RON knockdown and MSP (100 ng/ml) stimulation in PC3 and DU145 cells. (C and D) Transwell assays for the effects of RON silencing and MSP (100 ng/ml) on the migratory and invasive potentials of PC3 and DU145 cells. *P<0.05, **P<0.01, ***P<0.001; NS, not significant.

**Figure 4.**
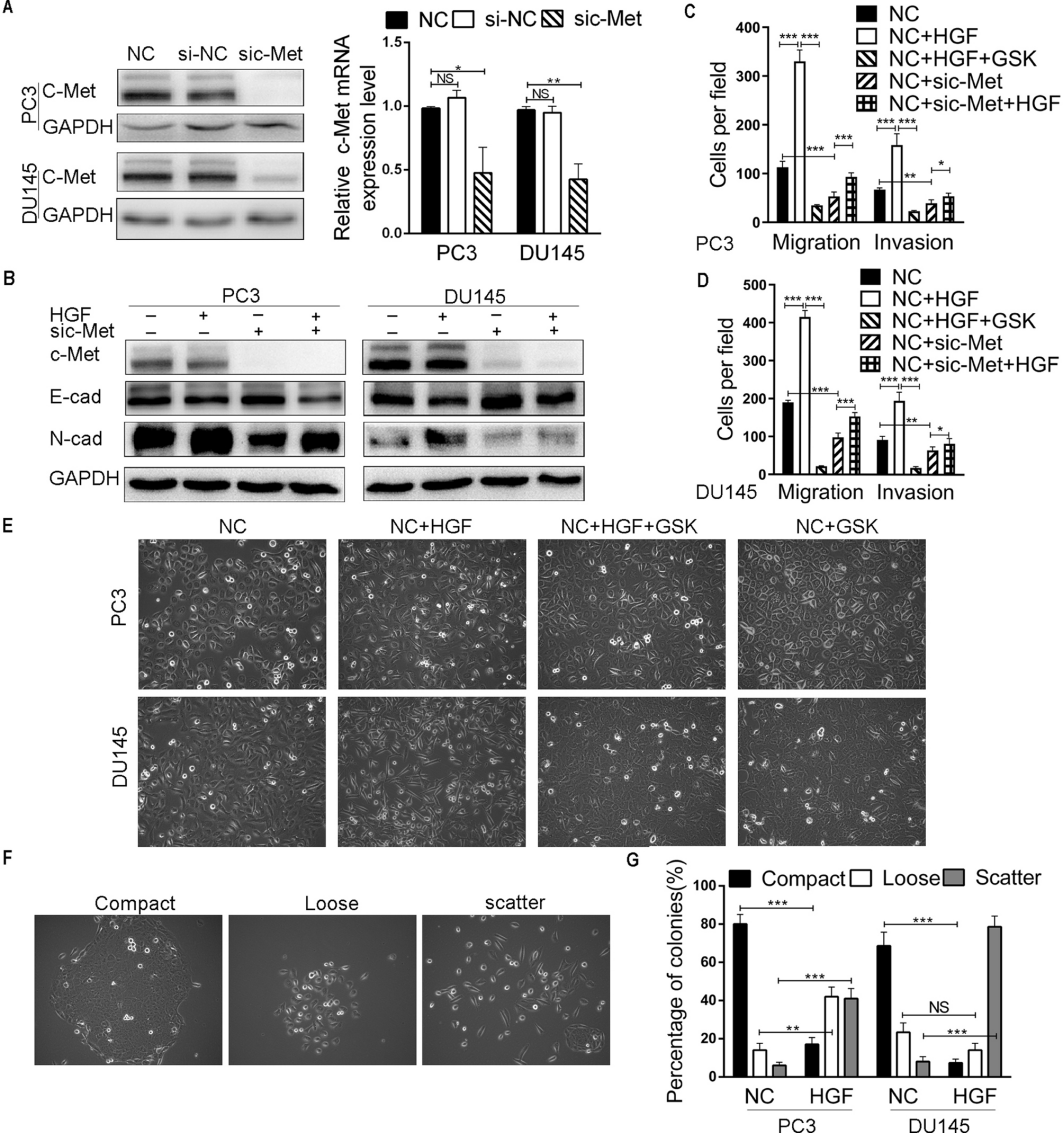
Functional role of c-Met in the metastasis and invasion of PCa cell lines. (A) Detection of the siRNA-mediated knockdown of c-Met in PC3 and DU145 cells by real-time PCR and western blotting. (B) The expression of c-Met, E-cadherin and N-cadherin was determined by western blotting after c-Met knockdown and HGF (50 ng/ml) stimulation in the PC3 and DU145 cells. (C and D) Transwell assays of the effects of c-Met silencing and HGF (50 ng/ml) on the migratory and invasive potentials of the PC3 and DU145 cells. (E) Morphological changes of PC3 and DU145 cells stimulated by HGF (50 ng/ml) and foretinib. (F and G) HGF (50 ng/ml) induced cell scattering in the PC3 and DU145 cells. *P<0.05, **P<0.01, ***P<0.001; NS, not significant.

**Figure 5.**
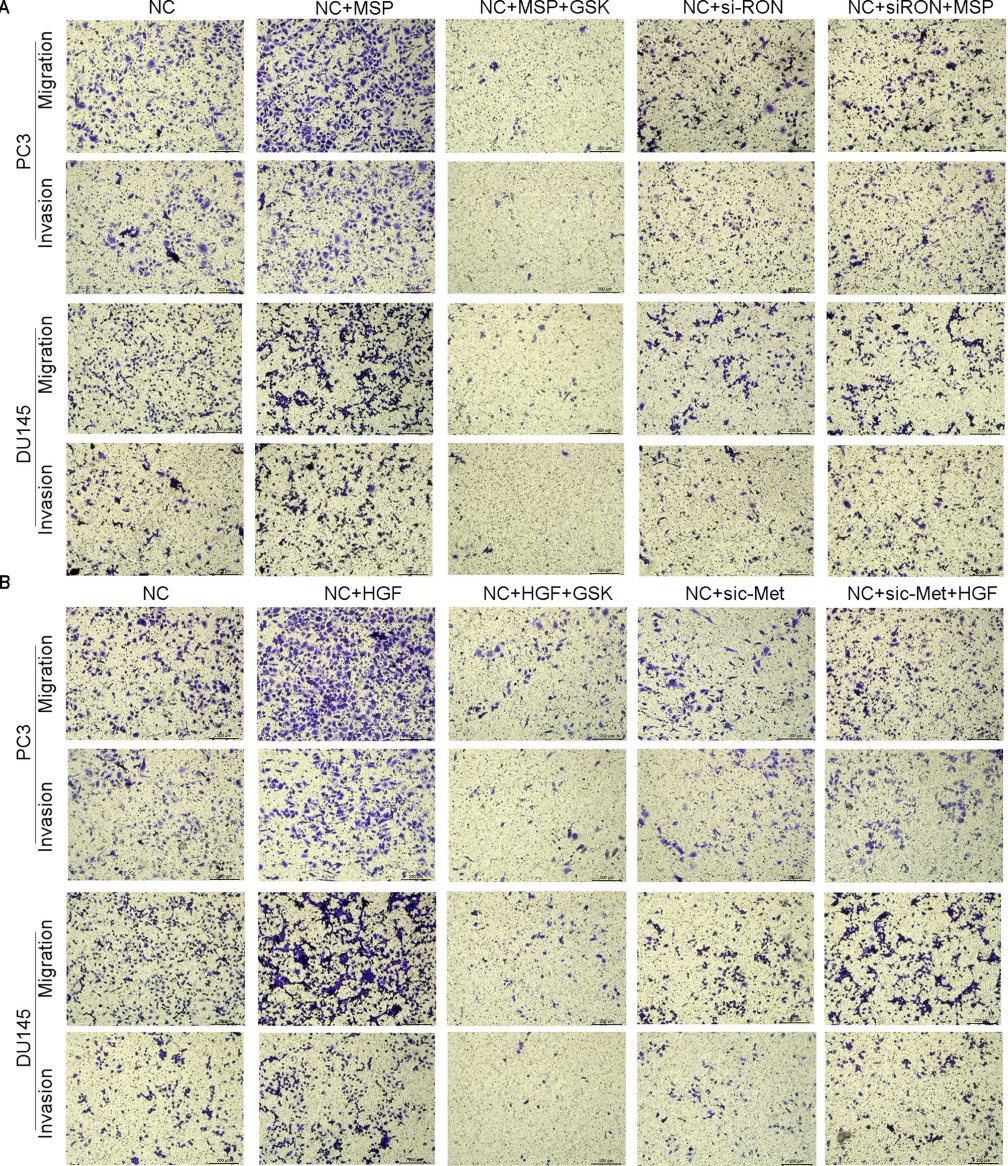
Functional role of RON and c-Met in the metastasis and invasion of PC a cell lines. (A) Transwell assays for the effects of si-RON and MSP (100 ng/ml) on migratory and invasive potentials of PC3 and DU145 cells (magnification, ×200). (B) Transwell assays for the effects of si-c-Met and HGF (50 ng/ml) on the migratory and invasive potentials of PC3 and DU145 cells (magnification, ×200).

**Figure 6.**
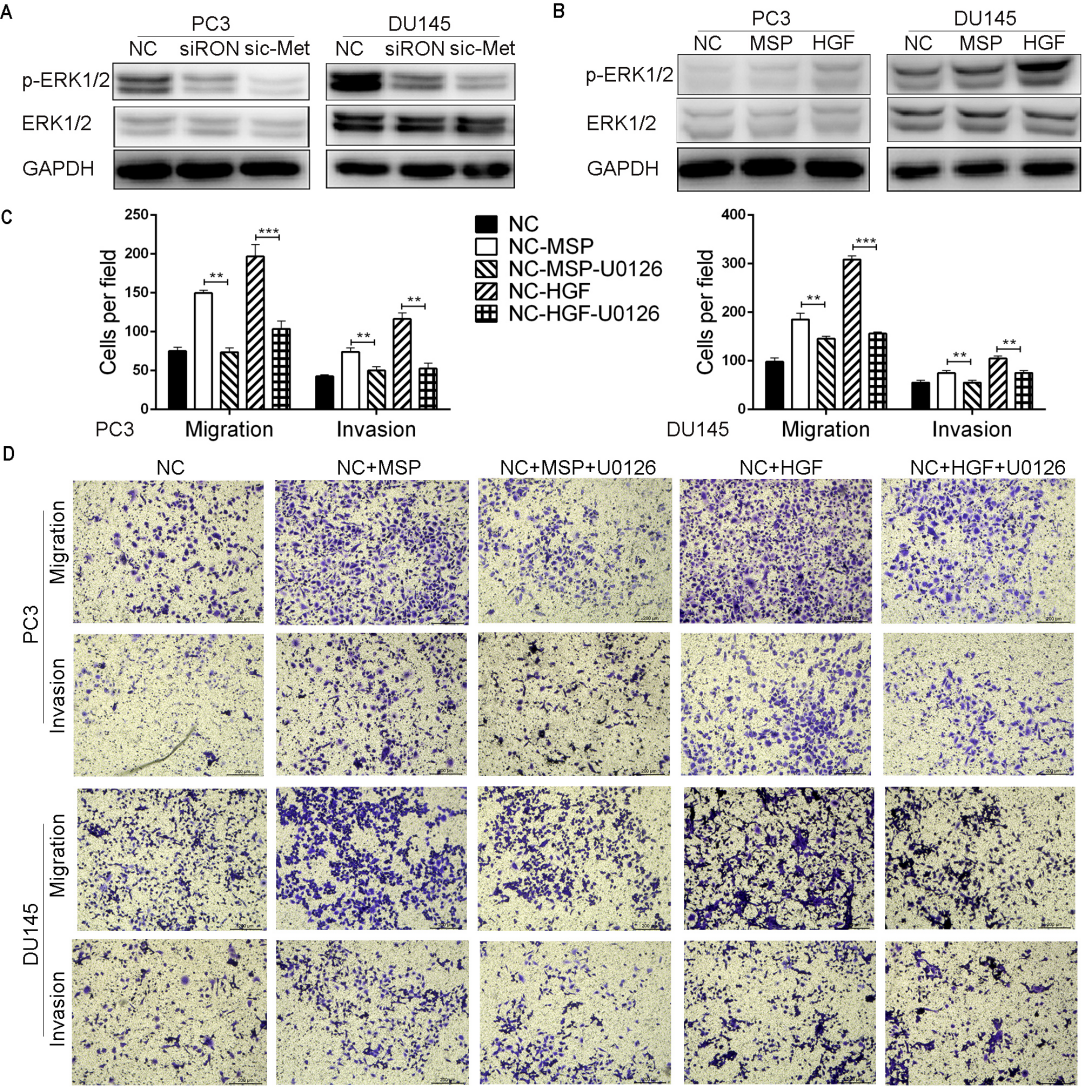
RON and c-Met mediates PCa metastasis via the ERK1/2 pathway. (A) Effect of RON and c-Met on phosphorylation of p-ERK1/2 was analyzed by western blotting. (B) PC3 and DU145 cells were treated with MSP or HGF for 24 h. Level of p-ERK1/2 was detected by western blotting. (C and D) Migration and invasion assays were carried out in the PC3 and DU145 cells following treatment with MSP, HGF and U0126 for 24 h (magnification, ×200). Each experiment was performed in triplicate. **P<0.01, ***P<0.001.

